# Inhibitory Impact of the Amino Benzoic Derivative DAB-2-28 on the Process of Epithelial–Mesenchymal Transition in Human Breast Cancer Cells

**DOI:** 10.3390/molecules30153284

**Published:** 2025-08-05

**Authors:** Laurie Fortin, Julie Girouard, Yassine Oufqir, Alexis Paquin, Francis Cloutier, Isabelle Plante, Gervais Bérubé, Carlos Reyes-Moreno

**Affiliations:** 1 Groupe de Recherche en Signalisation Cellulaire (GRSC), Département de Biologie Médicale, Université du Québec à Trois-Rivières, Trois-Rivières, QC G8Z 4M3, Canada; laurie.fortin2@uqtr.ca (L.F.); julie.girouard@uqtr.ca (J.G.); yassine.oufqir@signaturediscovery.com (Y.O.); 2 Regroupement Intersectoriel de Recherche en Santé de l’Université du Québec (RISUQ), Université du Québec, Québec, QC G1K 9H7, Canada; alexis.paquin@uqtr.ca (A.P.); francis.cloutier2@uqtr.ca (F.C.); isabelle.plante@inrs.ca (I.P.); 3 Groupe de Recherche en Signalisation Cellulaire (GRSC), Département de Chimie, Biochimie et Physique, Université du Québec à Trois-Rivières, Trois-Rivières, QC G8Z 4M3, Canada; 4 Institut National de la Recherche Scientifique (INRS)-Centre Armand Frappier Santé Biotechnologie, Laval, QC H7V 1B7, Canada

**Keywords:** inflammation, luminal breast cancer, macrophages, *para*-aminobenzoic acid (PABA), small molecules, triple-negative breast cancer

## Abstract

Macrophage-mediated inflammation is known to be involved in the epithelial–mesenchymal transition (EMT) of various types of cancer. This makes macrophage-derived inflammatory factors prime targets for the development of new treatments. This study uncovers the therapeutic potential and action mechanism of DAB-2-28, a small-molecule derived from *para-*aminobenzoic acid, in the treatment of breast cancer. The luminal MCF-7 and the triple-negative MDA-MB-231 cancer cell lines used in this study represent, respectively, breast cancers in which the differentiation states are related to the epithelial phenotype of the mammary gland and breast cancers expressing a highly aggressive mesenchymal phenotype. In MCF-7 cells, soluble factors from macrophage-conditioned media (CM-MØ) induce a characteristic morphology of mesenchymal cells with an upregulated expression of Snail1, a mesenchymal marker, as opposed to a decrease in the expression of E-cadherin, an epithelial marker. DAB-2-28 does not affect the differential expression of Snail1 and E-cadherin in response to CM-MØ, but negatively impacts other hallmarks of EMT by decreasing invasion and migration capacities, in addition to MMP9 expression and gelatinase activity, in both MCF-7 and MDA-MB-231 cells. Moreover, DAB-2-28 inhibits the phosphorylation of key pro-EMT transcriptional factors, such as NFκB, STAT3, SMAD2, CREB, and/or AKT proteins, in breast cancer cells exposed to different EMT inducers. Overall, our study provides evidence suggesting that inhibition of EMT initiation or maintenance is a key mechanism by which DAB-2-28 can exert anti-tumoral effects in breast cancer cells.

## 1. Introduction

Breast cancer (BC) is the most diagnosed and most lethal cancer afflicting women worldwide [1]. In reality, BC is a heterogeneous disease comprising multiple subtypes, each bearing different molecular characteristics, prognoses and therapeutical responses. They are characterized by the presence of certain cell surface receptors, such as the estrogen receptors (ER), the progesterone receptors (PR) and the human epidermal growth factor receptor 2 (HER2) [2]. They are generally divided into four major molecular subtypes: Luminal A and Luminal B (~30–70% of cases of breast cancers), HER2 tumors (~30%) and triple negative breast cancer (TNBC; ~15–20%), a BC subtype characterized by the lack of ER, PR and HER2 expression. Patients diagnosed with TNBC show increased recurrence and poor prognosis, compared to other subtypes [3]. TNBC are essentially unresponsive to current targeted therapies and are thus treated with conventional chemotherapeutic approaches. However, even with combined treatment regimens, survival rates remain low. Unfortunately, metastatic BC is associated with lethal outcomes and is generally considered incurable [4], the median survival of patients being less than one year [2,3]. Moreover, since the global incidence of BC is projected to rise by 38% by 2050 and its related deaths by 68% [5], novel and more effective compounds are critically needed to fight primary and metastatic BC.

Epithelial-to-mesenchymal transition (EMT) is a cellular program where epithelial cells lose their polarized structure and acquire a mesenchymal-like phenotype, enhancing their migratory and invasive capabilities [6]. In malignant diseases, this transformation is assumed to be a critical step in the progression of tumor cells from pre-invasive to invasive states and from organ-confined to metastatic disease [7]. During EMT, cancer cells are typically characterized by a decreased expression of E-cadherin (E-Cad) and by an increased expression of N-cadherin, vimentin and cellular proteases. Oncogenic EMT is thus associated with exacerbated cell motility, invasiveness, metastasis, and resistance to therapy [6,7].

Chronic inflammation [8] and EMT [9,10,11,12] are prime factors that enable BC progression and are strongly linked to the development of metastatic disease, which is the leading cause of death in BC patients [1,2,3,5]. Recent clinical and experimental data suggest that factors derived from tumor-associated macrophages (TAMs) play a crucial role in the regulation of EMT in BC [13,14,15,16,17,18]. Activated TAMs release cytokines and other factors that directly interact with BC cells, leading to increased cell adhesion, motility and invasion, which are all hallmarks of EMT [9,10,11,12]. TAMs, particularly M1-polarized macrophages (MØs), release high levels of the pro-inflammatory cytokines interleukin 6 (IL6) and tumor necrosis factor alpha (TNFα), which mediate the respective activation of Signal Transducer and Activator of Transcription 3 (STAT3) [15,16] and Nuclear Factor kappa-light-chain-enhancer of activated B cells (NFκB) signaling pathways [17,18]. In BC cells, the signaling pathways TNFα/NFκB and IL6/STAT3 are known to promote tumor progression via activation of key EMT-related transcription factors, such as Snail Family Transcriptional Repressor 1 (Snail1), Twist-related protein 1 (Twist1), and Zinc finger E-box binding homeobox 1 (ZEB1) [9,10,11,12,15,16,17,18]. These transcription factors are responsible for inducing the expression of so-called mesenchymal markers, while downregulating the expression of epithelial cell-specific proteins [6]. For instance, it has been shown that the TNFα/NFκB signaling pathway can play a pivotal role in the overexpression of matrix metalloproteases (MMPs), such as MMP2 and MMP9, as well as vimentin, a protein that composes the intermediate filaments of mesenchymal cells via the transcription factor Snail1 [19,20]. Also, it has been shown that an activated IL6/STAT3 signaling pathway inhibits the expression of E-Cad, a protein present in the adherens junctions of epithelia. In addition, this pathway induces the expression of vimentin and N-cadherin via the activation of the transcription factors Snail1 and Twist1 [21,22].

Therefore, therapeutic targeting of pro-tumor inflammatory mediators and signaling pathways presents a strong biological rationale for the development of new therapeutic approaches. In this optic, a synthetic *para*-aminobenzoic acid derivative, namely DAB-1, was initially identified by our laboratory as a molecule potentially used to target cancer-related inflammation [23,24]. In vivo studies using a murine model indicated that treatment with DAB-1 had no obvious effects on normal animal development and was associated with no signs of vital organ dysfunction, and that tumors are one of the main sites of DAB-1 accumulation. In addition, using preclinical models of murine bladder cancer, we demonstrated that repeated intraperitoneal injections of DAB-1 (150 μM) inhibited tumor growth by 90% and stopped the formation of pulmonary metastasis, likely by inhibiting the TNFα/NFκB and IL6/STAT3 signaling pathways [24].

**Figure 1:**
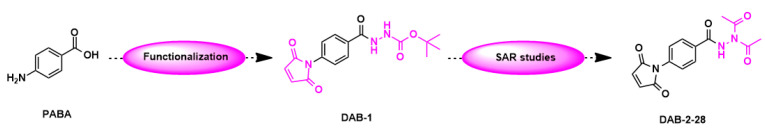
Structure of *para*-aminobenzoic acid (PABA), derivative DAB-1, and polyacetylated analog DAB-2-28.

In our quest for a more efficient cancer treatment, the structure of DAB-1 was further refined to provide a second-generation molecule named DAB-2-28 (Figure 1), with enhanced in vitro and in vivo biological properties compared to the original DAB-1 [25,26].

Data from in vitro studies revealed that DAB-2-28 displays less cytotoxic activity and greater efficiency than DAB-1 in inhibiting the production of nitric oxide (NO) as well as the activation of pro-inflammatory signaling pathways IL6/STAT3 and TNFα/NFκB. Moreover, while DAB-2-28 exhibited similar in vivo anti-inflammatory activity relative to DAB-1 in a model of carrageenan-induced acute inflammation, it efficiently inhibited the expression of the enzymes inducible nitric oxide synthase (iNOS) and cyclooxygenase-2 (COX-2) in peritoneal MØs. Notably, DAB-2-28 showed superior tumor development inhibition in models of murine bladder cancer. Thus, our studies provided preclinical proof-of-principle that DAB-2-28 is a suitable starting point in the treatment of cancer-related inflammation [25,26].

In this study, we hypothesize that DAB-2-28 could act on BC cells by inhibiting key inflammatory pathways involved in tumor progression and metastasis development. DAB-2-28 is foreseen to act on pathways activated by MØ-derived factors which promote the EMT process, as well as tumor migration and invasion.

## 2. Results

### 2.1. Impact of DAB-2-28 on the Viability and Proliferation of MCF-7 Cells Stimulated with Macrophage-Derived Factors

**Figure 2:**
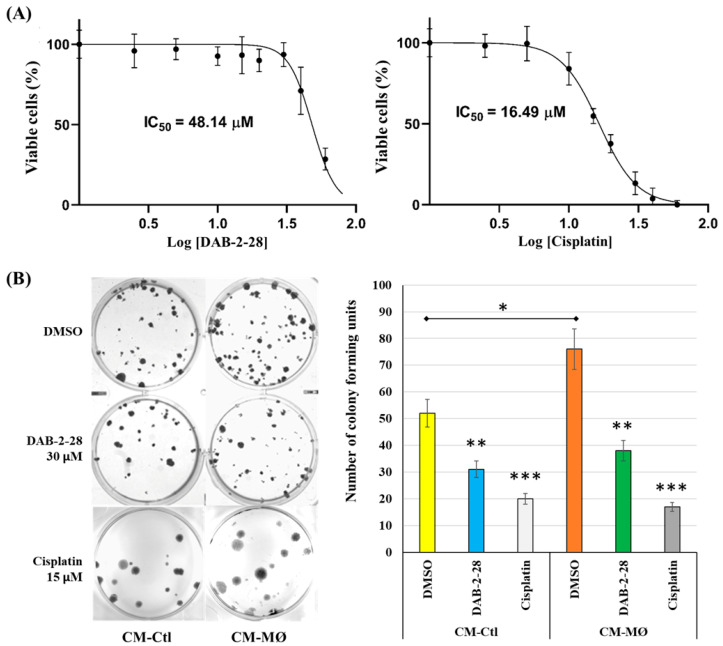
Effects of DAB-2-28 on MCF-7 cell viability and survival. (**A**) Representative dose–response curves using MTT cell proliferation assay and IC_50_ calculation, using different doses of DAB-2-28 or cisplatin (2.5–90 μM). (**B**) Representative images and graphical analysis of the number of colonies formed after 21 days of incubation. Colonies were stained with crystal violet and counted manually. * *p* < 0.05, ** *p* < 0.01 and *** *p* < 0.001 denote significant differences between the cell groups.

MCF-7 cells were treated with vehicle (0.1% DMSO in PBS) or increasing concentrations of DAB-2-28 or cisplatin for 24 h and their cytotoxicity was measured by an MTT assay. As shown in Figure 2A, DAB-2-28 and cisplatin reduced the viability of MCF-7 cells in a dose-dependent manner. The IC_50_ value of DAB-2-28 was determined to be 48.2 ± 1.9 μM. Cisplatin was used as a positive control and, as expected, its inhibitory effect was more potent than DAB-2-28, with an IC_50_ value of 16.5 ± 1.2 μM. Cell viability was about 77% at 30 μM for DAB-2-28 and about 80% at 15 µM for cisplatin, as compared to their controls. In subsequent experiments, BC cells were treated with a 30 μM dose of DAB-2-28, at which no obvious cell death was detected.

To further test whether DAB-2-28 suppresses growth of BC in response to treatment with MØ-derived factors, a colony formation assay was performed (Figure 2B). The colony formation assay (or clonogenic assay) is an in vitro cell survival assay based on the ability of a single cell to survive, proliferate and develop into a large colony through clonal expansion [27]. In this assay, MCF-7 cells were incubated for 48 h in CM-Ctl or CM-MØ before treatment with vehicle (0.1% DMSO in PBS), 30 μM DAB-2-28, or 15 μM cisplatin for 1 h, and cell survival was determined after 21 days of incubation (Figure 2B). To quantify the clonogenic activity of the cells, the colony was defined as a cell cluster of at least 50 cells, all originating from the division of an original single cell. The results demonstrate an increase in the number of colonies when the cells had been stimulated with CM-MØ, compared to CM-Ctl (Figure 2B). However, after DAB-2-28 pretreatment, the number of colonies formed by MCF-7 cells declined in both CM-Ctl and CM-MØ, with the decrease estimated at 40% and 51%, respectively (Figure 2B). By comparison with cisplatin, the number of colonies decreased by 62% and 78% when they were stimulated by CM-Ctl and CM-MØ. These data suggest that DAB-2-28 suppresses cell survival of MCF-7 cells, but with lower potency compared than cisplatin.

### 2.2. Effects of Macrophage-Derived Factors and DAB-2-28 on the Induction of EMT in MCF-7 Cells

It is well established that MØs play a major role in the tumor microenvironment by promoting cancer cell survival and invasion through the production of numerous inflammatory mediators; these mediators participate in extracellular matrix remodeling, angiogenesis and antitumor immune response suppression [13,14]. MØ-derived factors are also thought to act on cancer cells and to promote the typical features of EMT, including activation of pro-EMT transcription factors (Snail1, Twist1 and ZEB). Those proteins are associated with a loss of E-Cad expression, an increase in vimentin levels and a surge in the invasive tumoral potential via the expression of MMP2 and/or MMP9 [13,14]. To determine whether MØ-derived factors induce the EMT process in MCF-7 cells, we conducted a comparative study of soluble factors present in CM-Ctl and CM-MØ by evaluating their ability to cause morphological changes in the mesenchymal phenotype and to regulate the expression of E-Cad and Snail1.

#### 2.2.1. Induction of EMT by Macrophage-Derived Factors

**Figure 3:**
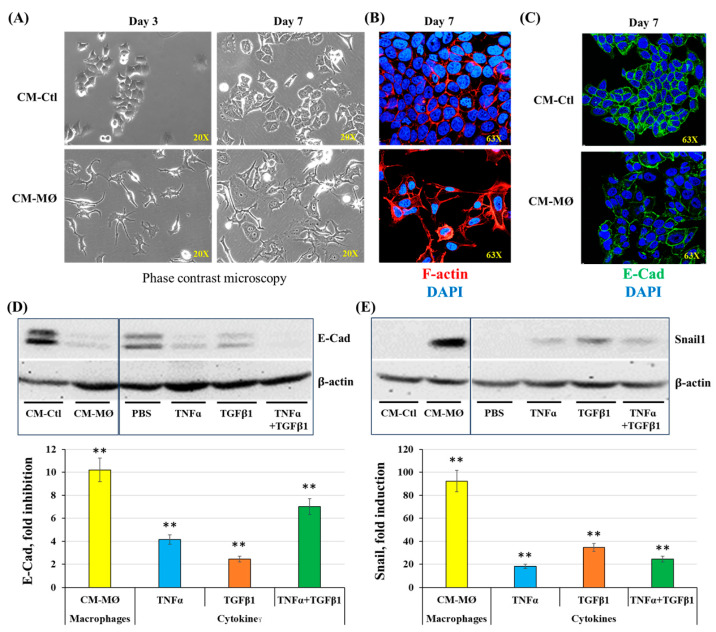
Effects of macrophage-derived factors on cell morphology and E-cadherin (E-Cad) and Snail1 expression by MCF-7 cells. (**A**,**B**) Representative images of cell morphology changes assessed by phase-contrast microscopy (days 3 and 7) and immunofluorescence (day 7) after cell incubation with CM-Ctl or CM-MØ. (**C**) E-Cad expression by immunofluorescence after incubation of cells with CM-Ctl or CM-MØ for 7 days. (**D**,**E**) Representative images and densitometric analyses of E-Cad and Snail1 expression in MCF-7 cells stimulated with controls (CM-Ctl or PBS), CM-MØ, TNFα, TGFβ1 or TNFα + TGFβ1 for 7 days (for E-Cad) or 6 h (for Snail1). Data are expressed as fold induction or fold inhibition compared to controls. ** *p* < 0.01 denotes significant differences compared to controls (CM-Ctl or PBS).

In a first series of experiments, MCF-7 cells were stimulated with CM-Ctl or CM-MØ for 7 days and cell morphology was monitored throughout incubation. Pictures were taken under a phase-contrast microscope on days 3 and 7 (Figure 3A). Data obtained by phase contrast microscopy and immunofluorescence indicate that MCF-7 cells adopt a different morphology when incubated with CM-MØ. The cells were found to adopt irregular and elongated shapes with several cytoplasmic extensions, which are prime characteristics of mesenchymal cells [28]. In contrast, cells incubated with CM-Ctl maintained a rather regular and rounded shape, characteristic of epithelial cells (Figure 3A). On day 7 of incubation, the organization of actin filaments was analyzed by immunofluorescence labeling with phalloidin (Figure 3B; red staining). Images obtained by fluorescence microscopy show that the conformation of actin filaments was disrupted when MCF-7 cells were incubated with CM-MØ. When incubated with CM-Ctl, actin filaments were arranged at the cell junctions surrounding the plasma membranes (Figure 3B). However, when cells were incubated with CM-MØ, actin filaments formed a network of stress fibers like those present in mesenchymal cells [7], as observed in the presence of a fiber cluster in the cytoplasm of MCF-7 cells (Figure 3B; F-actin).

The expression of epithelial and mesenchymal markers was studied by immunofluorescence (IF) and Western blot (WB) techniques in the MCF-7 cells stimulated with CM-Ctl or CM-MØ. To validate and further support these results, cells were also stimulated with three MØ-derived factors known to induce EMT, namely, IL6, TNFα and transforming growth factor beta 1 (TGFβ1) [15,16,17,18].

Regarding the epithelial marker E-Cad, IF microscopy images show a marked decrease in E-Cad expression at the plasma membranes of MCF-7 cells stimulated with CM-MØ, compared to CM-Ctl (Figure 3C). Results from WB analysis indicate that soluble factors from CM-MØ significantly inhibit E-Cad expression in MCF-7 cells by approximately 10-fold (*p* < 0.01) compared to stimulation with CM-Ctl. However, when compared to PBS, the inhibition rates of E-Cad were determined to be 4-fold, 2-fold and 7-fold when cells were, respectively, stimulated by TNFα, TGFβ1 and by the combination of both cytokines (Figure 3D). In contrast to E-Cad, Snail1 expression was increased by approximately 92-fold when MCF-7 cells were stimulated with CM-MØ, compared to CM-Ctl (Figure 3E). In cells exposed to PBS and cytokines with pro-EMT activity, induction rates were determined to be 18-fold, 34-fold and 24-fold when the cells were stimulated with TNFα, TGFβ1, and TNFα plus TGFβ1 (Figure 3E).

#### 2.2.2. Effect of DAB-2-28 on Cellular Features Associated with EMT

**Figure 4:**
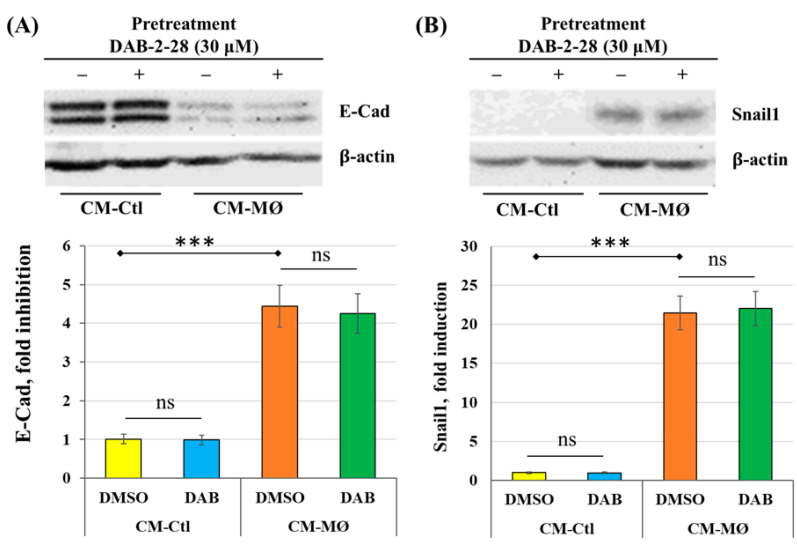
Effects of DAB-2-28 on Snail1 and E-Cad expression induced by macrophage-derived factors in MCF-7 cells. Representative images and graphical analyses of E-Cad (**A**,**B**) Snail1 expression observed by Western blot in MCF-7 cells pretreated with 30 μM DAB-2-28 for 1 h and stimulated with CM-MØ for 7 days to assess E-Cad expression, or for 6 h to assess Snail1 expression. *** *p* < 0.001 denotes a significant difference between CM-Ctl and CM-MØ; ns: non-significant difference for DAB-2-28 compared to DMSO.

To determine whether DAB-2-28 affects the cellular characteristics typically associated with the EMT process induced by MØ-derived factors, biochemical and biological analyses were performed to study (a) the expression of Snail1 and E-Cad, by WB; (b) cell invasion, using a Boyden chamber microinvasion assay; (c) cell motility, by a wound healing assay; (d) activation and expression of the metalloproteinase MMP9, using gelatin zymography and WB; and (e) induction of signaling pathways involved in tumor progression and EMT, by WB. As previously observed, soluble factors from CM-MØ induced a significant decrease (*p* < 0.01) in E-Cad expression (Figure 4A) as well as a strong expression of Snail1 (Figure 4B) in DMSO-pretreated MCF-7 cells. However, the differential expression of E-Cad and Snail1 proteins induced by factors present in CM-MØ was not affected in cells pretreated with DAB-2-28 (Figure 4).

**Figure 5:**
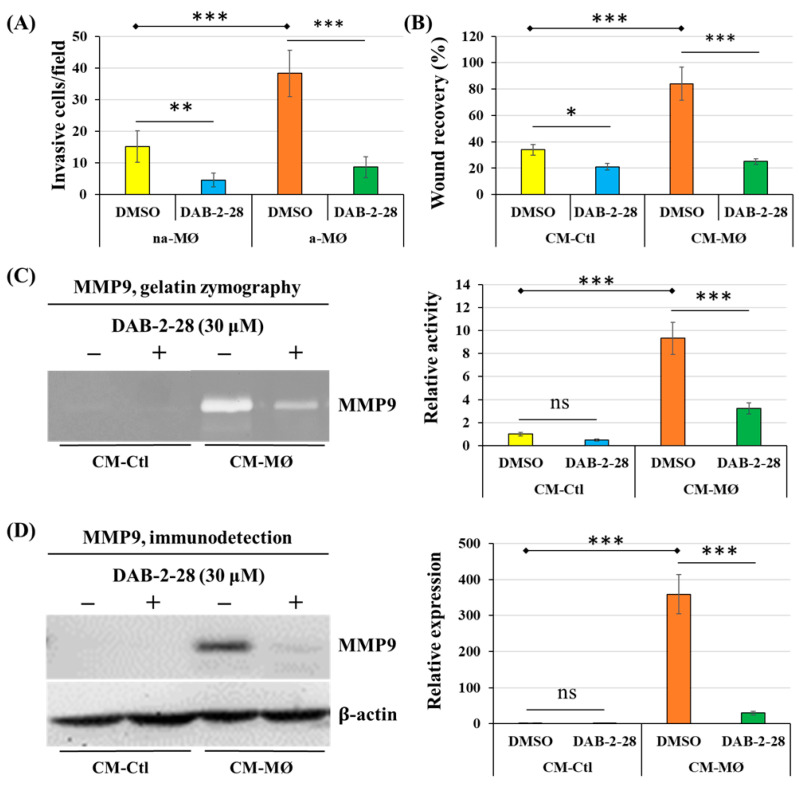
Effects of DAB-2-28 on the invasive potential and motility of MCF-7 cells stimulated by macrophage-derived factors. (**A**) Graphical analysis represents the number of invasive cells per field, when cells were pretreated for 1 h with DMSO or DAB-2-28 (30 μM) and stimulated in coculture with non-activated (na-MØ) or activated (a-MØ) macrophages for 48 h. (**B**) Graphical analysis of cell migration assays performed with MCF-7 cells stimulated with CM-Ctl or CM-MØ for 48 h, and then treated with DMSO or DAB-2-28 (30 μM) for 1 h. (**C**,**D**) Representative images and graphical analyses of gelatin zymography and Western immunoblotting assays used to evaluate, respectively, the activation and the expression of matrix metalloproteinase MMP9. MCF-7 cells were pretreated with DMSO or DAB-2-28 (30 μM) for 1 h and then stimulated with CM-Ctl or CM-MØ for 24 h. * *p* < 0.05, ** *p* < 0.01 and *** *p* < 0.001 denote significant differences between the cell groups. ns: not significant.

Next, a series of experiments were performed to evaluate the impact of DAB-2-28 on the invasive potential acquired when epithelial-phenotype MCF-7 cancer cells undergo EMT in response to MØ-derived factors (Figure 5). First, invasion assays were performed by coculturing MCF-7 cells with non-activated (na-MØ) or activated (a-MØ) MØs to allow paracrine interactions between these populations. To quantify the invasive activity of MCF-7 cells, we counted the number of invasive cells per field, depending on the culture and pretreatment conditions. Without DAB-2-28 pretreatment, the number of invasive MCF-7 cells was approximately 2.5 times greater when cocultured with a-MØ than when cocultured with na-MØ, as reported in Figure 5A. In contrast, when pretreatments with 30 μM DAB-2-28 were performed, the number of invasive MCF-7 cells significantly decreased (Figure 5A).

Migration assays were performed with MCF-7 cells cultured in CM-Ctl or CM-MØ for 48 h before treatment with vehicle (0.1% DMSO) or DAB-2-28 (30 μM). To quantify the motility/migration properties of MCF-7 cells, we evaluated the percentage of wound closure. This percentage was calculated from the wound areas measured based on images taken at the initial (t = 0 h) and final (t = 24 h) time-points of the experiment. The results reported in Figure 4B show that, without DAB-2-28 pretreatment, MCF-7 cells were more motile when stimulated with CM-MØ, compared to CM-Ctl, with wound closures at t = 24 h determined to be 83%. In contrast, pretreatment with DAB-2-28 at 30 µM significantly decreased the wound closure ability of CM-MØ-stimulated MCF-7 cells, with wound closure percentages at t = 24 h being 27% (Figure 5B).

To further document the effect of DAB-2-28 on the invasive potential of MCF-7 cells in response to MØ-derived factors, the activity of secreted MMP9 proteinase was studied using gelatin zymography (Figure 5C) and its expression was analyzed by WB (Figure 5D). An increase in MMP9 gelatinase activity was observed when cells were stimulated with CM-MØ, while it was decreased by 30 μM with DAB-2-28 treatment (Figure 5C). Similarly, an increase in MMP9 protein expression was induced when cells were stimulated with CM-MØ, and this inductive effect was inhibited when they were pretreated with 30 μM DAB-2-28 (Figure 5D).

**Figure 6:**
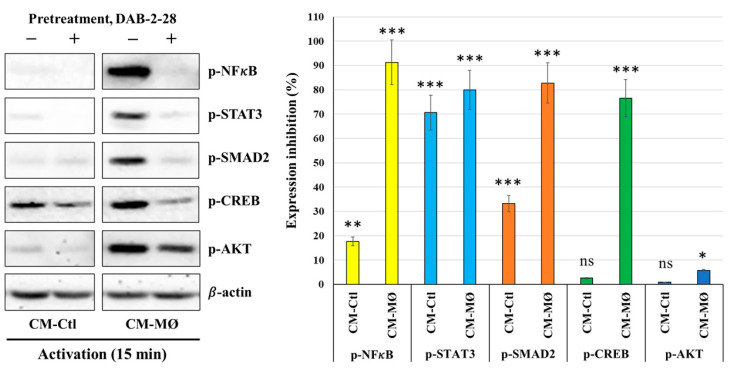
Effect of DAB-2-28 on the activation of pro-tumor signaling pathways induced by macrophage-derived factors in MCF-7 cells. Representative images and graphical analyses of selected pro-tumor and pro-TEM signaling pathways immunodetected by WB. Cells were pretreated with DAB-2-28 (30 μM) or DMSO for 1 h and activated with CM-Ctl or CM-MØ for 15 min. The expression levels of phosphorylated (p-) form relative to the total form for each protein were evaluated. The graph illustrates the relative expression inhibition for each phosphorylated protein induced under DAB-2-28 treatment, compared to DMSO. Representative images of total protein expression are not shown, to simplify the figure. β-actin was used as a loading control. * *p* < 0.05, ** *p* < 0.01 and *** *p* < 0.001 denote significant differences between control DMSO (-) and DAB-2-28 (+). ns: not significant.

Finally, a cell signaling study was performed to better understand the antitumoral action mechanism of DAB-2-28 on EMT initiation in MCF-7 cells in response to MØ-derived factors (Figure 6). In this study, cells were first pretreated with DAB-2-28 (30 μM) or DMSO for 1 h and then stimulated with CM-Ctl or CM-MØ for 15 min. The data reported in Figure 6 show that the phosphorylation levels of NF-κB, STAT3, SMAD2 (Mothers against decapentaplegic homolog 2), CREB (cAMP response element-binding protein) and AKT (Protein Kinase B) were significantly increased when MCF-7 cells were subjected to 15 min activation by CM-MØ. Notably, this increase in the activation of pro-TEM signaling proteins was significantly inhibited when cells were pretreated with DAB-2-28. The final results show relatively high levels of the active/phosphorylated form of CREB protein in both CM-Ctl- and CM-MØ-stimulated MCF-7 cells. However, the levels of the active/phosphorylated form of CREB protein diminished after pretreatment with DAB-2-28 and activation with both CM-Ctl and CM-MØ (Figure 6).

### 2.3. Induction of EMT in MCF-7 Cells by the StemXvivo Reagent

**Figure 7:**
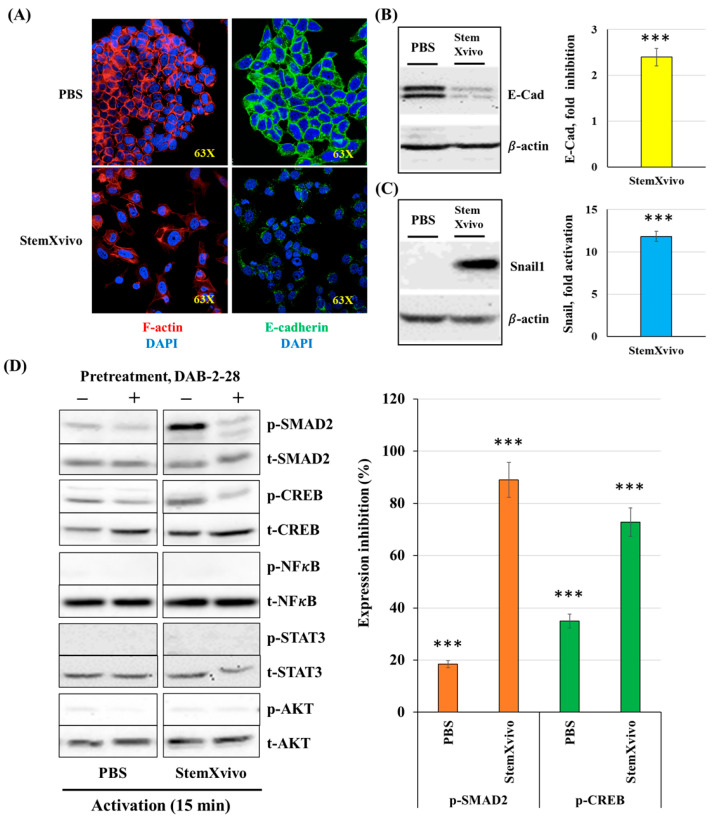
Effect of DAB-2-28 on StemXvivo-induced TEM parameters in MCF-7 cells. (**A**) Representative images of F-actin (phalloidin; red), and E-Cad expression, assessed by immunofluorescence after cell incubation with StemXvivo reagent. (**B**,**C**) Representative images and graphical analyses of E-Cad and Snail1 expression in MCF-7 cells stimulated with PBS or StemXvivo reagent. Cells were incubated for 6 h for Snail1 and 7 days for E-Cad. Data are expressed as fold inhibition or fold induction, compared to PBS. *** *p* < 0.001; StemXvivo versus PBS. (**D**) Representative images and graphical analyses showing the effect of DAB-2-28 on StemXvivo-induced activation of pro-tumor signaling pathways in MCF-7 cells. The graph represents the relative expression inhibition for each phosphorylated protein induced under DAB-2-28 treatment, compared to DMSO. Phosphorylated proteins for which no induction was observed are not plotted. *** *p* < 0.001 denotes significant differences between control DMSO (-) and DAB-2-28 (+).

Our results indicate that MØ-derived factors induce TEM in MCF-7 cells, but the DAB-2-28 molecule did not affect the expression of Snail1 and E-Cad. However, this small molecule can inhibit other parameters related to EMT and tumor aggressiveness, such as invasion and pro-tumor signaling pathways. Subsequently, we opted for an alternative method of EMT induction using the StemXvivo^®^ EMT Inducing Media reagent. The objective of the experiments performed was twofold: determine which pathways are activated by the StemXvivo reagent and which are inhibited by treatment with DAB-2-28. The StemXvivo reagent is a culture medium supplement containing several factors, including antibodies against human E-Cad protein and human TGFβ cytokine. It has been designed to induce EMT in several cell lines, including MCF-7 cells [29]. First, we confirmed the ability of this reagent to induce EMT in MCF-7 cells by observing the presence of E-Cad, along with changes in the conformation of actin filaments (phalloidin labeling), detected by immunofluorescence (Figure 7A). Variation in the expression levels of Snail1 and E-Cad were evaluated by WB (Figure 7B,C). Microscopy-based image analysis showed a decline in E-Cad expression by immunofluorescence as well as a change in the conformation of actin filaments in cells stimulated with the StemXvivo reagent (Figure 7A). In addition, WB analyses revealed an increase in Snail1 expression and a decrease in E-Cad expression (Figure 7B,C). Regarding signaling studies, MCF-7 cells were pretreated for 1 h with 30 μM DAB-2-28 before being subjected to a 15 min stimulation with StemXvivo (Figure 7D). The study was limited to the immunodetection of proteins, in the phosphorylated form, aiming to detect the most relevant pro-TEM signaling proteins, including NFκB, STAT3, SMAD2 and CREB. The results, reported in Figure 7D, show low or no detection of NFκB, STAT3, or AKT proteins in the active form under both conditions, but an increase of the active form of the SMAD2 and CREB proteins when promoted by the StemXvivo reagent was observed. Notably, the densitometry analysis shows that DAB-2-28 inhibits the activation of the SMAD2 and CREB proteins when induced by the StemXvivo reagent (Figure 7D).

### 2.4. Inhibition of Migration, Invasion, and MMP9 Activity with Associated Signaling Pathway Modulation in Triple-Negative MDA-MB-231 Breast Cancer Cells 

**Figure 8:**
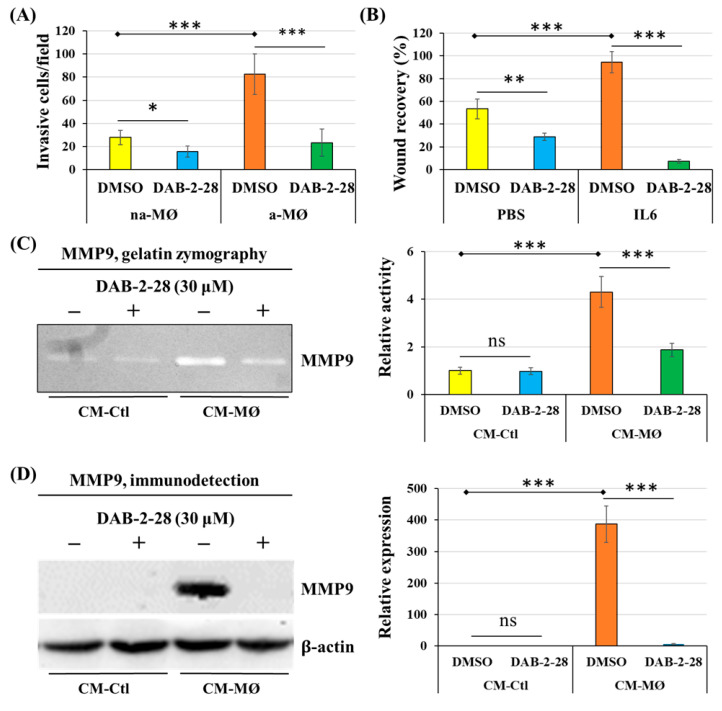
Effects of macrophage-derived factors and DAB-2-28 on cell invasion and motility, as well as on MMP9 activation and expression in MDA-MB-231 cells. (**A**) Graphical analysis representing the number of invasive cells per field, when cells were pretreated for 1h with DMSO or DAB-2-28 (30 μM) before stimulation in coculture with non-activated (na-MØ) or activated (a-MØ) macrophages for 48 h. (**B**) Graphical analysis of cell migration assays performed with MDA-MB-231 cells stimulated with 25 ng/mL IL-6 for 24 h, and then treated with DMSO or DAB-2-28 (30 μM) for 1 h. (**C**,**D**) Representative images of gelatin zymography and Western immunoblotting assays to assess, respectively, the activation and the expression of matrix metalloproteinase MMP9. MDA-MB-231 cells were pretreated with DMSO or DAB-2-28 (30 μM) for 1 h and stimulated with CM-Ctl or CM-MØ for 24 h. * *p* < 0.05, ** *p* < 0.01 and *** *p* < 0.001 denote significant differences between the cell groups. ns: not significant.

To test the antitumoral properties of DAB-2-28 on cells with a mesenchymal phenotype, several experiments were performed using a triple-negative MDA-MB-231 breast cancer cell line. First, we determined the effect of DAB-2-28 on cell invasion, using a Boyden chamber invasion assay (Figure 8A), and cell motility, using a wound closure migration assay (Figure 8B). The results, reported in Figure 8A, show that coculture with activated MØs (a-MØ) increases the number of invasive cells per field, compared to coculture with non-activated MØs (na-MØ). Furthermore, pretreatment with 30 μM DAB-2-28 efficiently decreased the number of invasive cells when MDA-MB-231 cells were cocultured with both, na-MØs and a-MØs (Figure 8A).

Our previously published studies demonstrated that activation of the IL6/STAT3 pathway plays a major role in the induction of cell motility, especially in MØs and bladder cancer cells [23]. The results reported in Figure 8B confirm that stimulation with IL6 induces migration of MDA-MB-231 cells, with approximately 90% wound closure after 24 h of incubation with IL6, compared to 50% with PBS. However, MDA-MB-231 cells become less motile and less efficient in closing wounds following DAB-2-28 treatment; the wound closure level is approximately 30% when incubated with PBS, and 10% with IL6 (Figure 8B).

Next, to better understand the inhibitory mechanism of DAB-2-28 in its effect on the invasive capacity of MDA-MB-231 cells, we quantified MMP9 activation (Figure 8C) and expression (Figure 8D). The gelatin zymography results indicate that stimulation of MDA-MB-231 cells with CM-MØ significantly increases MMP9 gelatinase activity, compared to stimulation with CM-Ctl. In addition, pretreatment with DAB-2-28 at 30 μM decreases the activity of MMP9 secreted by MDA-MB-231 cells stimulated with CM-MØ (Figure 8C). The WB results demonstrate that MMP9 expression is significantly induced by CM-MØ stimulation and that this effect was completely inhibited when cells were pretreated with 30 μM DAB-2-28 (Figure 8D).

**Figure 9:**
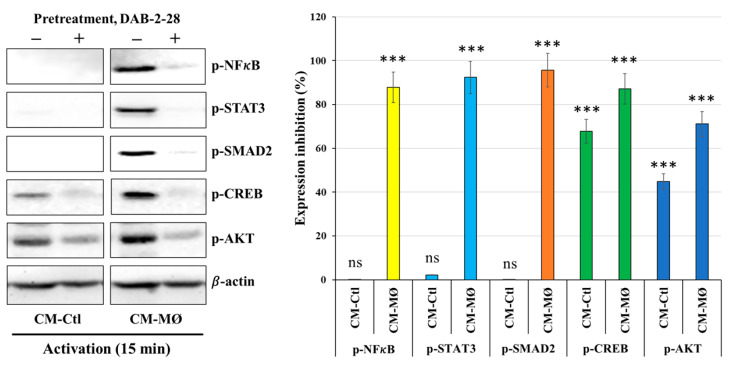
Effects of macrophage-derived factors and DAB-2-28 on the activation of pro-tumor signaling pathways in MDA-MB-231 cells. Representative images and graphical analyses of selected pro-tumor signaling pathways immunodetected by WB. Cells were pretreated with DAB-2-28 or DMSO for 1 h before activation with CM-Ctl or CM-MØ for 15 min. The relative expression levels of phosphorylated (*p*-) form against total form were evaluated for each protein. The graph represents the relative expression inhibition for each phosphorylated protein induced under DAB-2-28 treatment, compared to DMSO. Representative images of total protein expression are not shown, to simplify the figure. β-actin was used as a loading control. *** *p* < 0.001 denote significant differences between control DMSO (-) and DAB-2-28 (+). ns: not significant.

Finally, a signaling study was performed to determine which signaling pathways are affected by DAB-2-28 in MDA-MB-231 cells stimulated with CM-Ctl or CM-MØ. The WB results reported in Figure 8 indicate that the expression levels of the phosphorylated forms of NFκB, STAT3, SMAD2, CREB and AKT are upregulated upon activation with CM-MØ, compared to CM-Ctl. Furthermore, DAB-2-28 pretreatment impeded the expression of these phosphorylated proteins. Analysis of CREB and AKT proteins indicates that they are expressed in their active and phosphorylated form in both CM-Ctl- and CM-MØ-stimulated cells. However, CREB and AKT protein activation levels are greatly inhibited by pretreatment with 30 μM DAB-2-28 (Figure 9).

## 3. Discussion and Conclusions

This study aims to demonstrate that DAB-2-28 acts on breast cancer cells by inhibiting key inflammatory pathways involved in tumor progression and metastasis. An important aspect of this research program was the elucidation of the regulatory mechanism of DAB-2-28; the compiled results highlight its chemopreventive potential against breast cancer cells. Indeed, this small molecule appears to act on signaling pathways activated by MØ-derived factors that promote the EMT process.

Our results also indicate that DAB-2-28 does not completely reverse the EMT process induced by MØ-derived factors, as it was unable to inhibit the gain of Snail1 expression in MCF-7 cells. Thus, the discovery that E-Cad expression is not restored by DAB-2-28 is not surprising given that Snail1 is the strongest repressor of E-Cad [8,9]. The reason why Snail1 expression was not inhibited is less clear, as our results show that DAB-2-28 can inhibit several signaling pathways responsible for inducing the expression of Snail1, namely, TGFβ/SMAD2, IL6/STAT3 and TNFα/NFκB [8,9]. It is possible that the expression of Snail1 is induced by a pathway that is not inhibited, or not completely inhibited, by DAB-2-28. To further understand the regulation of Snail1 in this context, various inhibitors for specific pathways could be used to investigate the expression or the intracellular localization of Snail1. Alternatively, higher doses of DAB-2-28 could also be employed to verify whether incomplete inhibition of certain pathways might be responsible for the sustained Snail1 expression in response to pro-EMT-derived factors.

However, it is important to note that in the context of cancer, EMT presents itself as a spectrum with various transient states, meaning that cancer cells often undergo only a partial EMT and, as a result, display both epithelial and mesenchymal markers [28,30]. Since EMT is a complex process regulated by many interdependent pathways and transcription factors [30], targeting EMT for the development of cancer therapies is more complicated than merely the inhibition of Snail1 expression or the restoration of E-Cad expression. In fact, studies have shown that even completely silencing Snail1 does not revert mesenchymal state breast cancer cells back to an epithelial state, nor does it restore E-Cad expression in cells [31]. Importantly, partial reversion of EMT has also been described in the literature. Using U0126 and PD98059 inhibitors, a study conducted by Li and Mattingly reported that inhibition of ERK MAPK kinase activation promotes the reversion to epithelial morphology in Ras-transformed breast epithelial cells [32]. However, PD184352, which was more effective than U0126 in the reversion to normal epithelial morphology, did not increase the expression of E-Cad, suggesting that MAPK kinase inhibitors are effective in restoring epithelial morphology in the absence of a significant effect on E-cadherin expression levels [32]. Considering this observation, we believe inhibiting the functional properties acquired by cancer cells following EMT is of the utmost importance for development of new cancer therapies, whether it be dependent upon or independent of the expression of epithelial/mesenchymal cellular markers.

In this context, we have shown that DAB-2-28 negatively regulates the migration and invasion capacities of both MCF-7 and MDA-MB-231 cells in response to MØ-derived factors, most probably by decreasing MMP9 gelatinase activity and inhibiting activation of the key pro-EMT proteins NFκB, STAT3, AKT and SMAD2. In fact, by degrading collagens, fibronectin, laminin and other structural proteins of the extracellular matrix, MMPs play a critical role in mediating EMT and thus stimulating tumor promotion and metastasis [33]. As MMPs are considered attractive therapeutic targets, numerous promising small-molecule MMP inhibitors demonstrating varying degrees of potency and selectivity have been discovered [34]. However, while some of them advanced into clinical trials, they were generally unsuccessful due to poor oral bioavailability, low selectivity among MMP isoforms, or intolerable side effects in part due to simultaneous inhibition of non-target metalloproteases. Small molecules containing a marcaptoacyl function for MMP catalytic zinc chelation, such as rebimastat (BMS-275291), constitute an example of a promising MMP2/MMP9 inhibitor that failed to be incorporated into adjuvant therapy due to severe musculoskeletal toxicity, which was observed in a Phase II trial for early breast cancer [35].

While promising, the development of safe and effective EMT-targeting drugs is still an active area of research. Based on our results, we believe DAB-2-28 deserves more scientific attention due to its potent inhibitory activity against multiple signaling pathways and favorable safety profile. Further studies using human tumor xenograft should include pharmacokinetic and toxicokinetic assessments to determine the maximum tolerated dose of this compound and the identification of target organs to ultimately improve the safety of long-term treatments, which could be extended to different types of cancer.

## 4. Materials and Methods

### 4.1. Experimental Models

The human breast cancer cell lines MCF-7 and MDA-MB-231, as well as the human monocyte cell line THP-1, were purchased from ATCC (American Type Culture Collection). The THP-1 cell line is a model widely used to represent MØs derived from blood monocytes [23]. Among human breast cancer cell lines, MCF-7 cells are used in breast cancer studies to represent luminal A cancers, differentiated cancer cells that resemble the healthy phenotype of a mammary gland epithelial cell. The MDA-MB-231 cell line represents a more aggressive and invasive TNBC. These cells are highly dedifferentiated and resemble a mesenchymal phenotype [36,37]. They are employed to mimic cells that have undergone dedifferentiation toward a mesenchymal phenotype.

### 4.2. Cell Culture and Reagents

The cells were cultured in a complete culture medium consisting of RPMI-1640 medium supplemented with 10% heat-inactivated fetal bovine serum (FBS), 1 mM sodium pyruvate, 10 mM HEPES, and 50 μg/mL gentamicin. The cells were kept in a humidified incubator at 37 °C with 5% CO_2_. All culture media, in addition to the HBSS (Hank’s Balanced Salt Solution), PBS (Phosphate-buffered saline), serum, and reagents, were purchased from Wisent Bioproducts (Saint-Bruno, QC, Canada). As previously described [25,26], DAB-2-28 was synthetized using DAB-1 as the starting material in a three-step reaction sequence. DAB-2-28 was initially dissolved in 100% dimethyl sulfoxide (DMSO; Chemical Company, Oakville, ON Canada Sigma) to prepare DAB-2-28 stock solutions, which were 1000-fold diluted in PBS before use. Then, a solution of 0.1% DMSO in PBS was used as vehicle.

### 4.3. Production of Macrophage Conditioned Media

Conditioned media from non-activated and activated MØs were produced to stimulate cancer cells in several experiments. First, to differentiate THP-1 monocytes into MØs, the cells were placed in a 6-well plate (3 × 10^6^ cells/well) and treated with 80 ng/mL PMA (phorbol-12-myristate-13-acetate; Sigma-Aldrich, Saint Louis, MO, USA) in complete culture medium for 48 h. Subsequently, MØs were polarized toward a pro-inflammatory phenotype with complete culture medium containing 5 ng/mL of interferon gamma (IFN-γ) and 25 ng/mL of TNF-α (Peprotech Inc., Montreal, QC, Canada) for 24 h. For non-activated MØs, the PMA-containing culture medium was replaced with fresh complete culture medium (without PMA and cytokines). Then, the culture medium was changed to fresh, serum-free medium, in which the cells were incubated for 48 h. After incubation, the supernatant was harvested, yielding the conditioned medium of activated MØs (CM-MØ) and the conditioned medium of non-activated MØs, the latter of which served as a control (CM-Ctl).

### 4.4. Cell Viability Assay (MTT Assay)

MTT assays were performed to evaluate the effects of DAB-2-28 on cell viability in MCF-7 cells at different concentrations, as previously described. Briefly, cells were plated in 96-well plates (5 × 10^3^ cells/well) and left to adhere overnight. The next day, they were treated with DMSO, DAB-2-28, or cisplatin for 24 h, at concentrations ranging from 2.5 to 90 μM, in complete culture medium. Then, the MTT dye (tetrazolium 3,4,5 dimethylthiazol-2-yl)-2,5-diphennyltetrazolium bromide; Sigma Chemical Company, Oakville, ON, Canada) was added to each well to obtain a final concentration of 0.5 mg/mL, and the plate was incubated for 3 h. During this incubation period, the viable cells reduced MTT to purple formazan crystals, which are insoluble in aqueous media. After 3 h of incubation, the supernatant was aspirated and 100 μL of an acidic isopropanol-based solubilization solution was added to each well, solubilizing the crystals and producing a homogeneous solution, the blue color intensity of which was quantified by colorimetry using a spectrophotometer (Biotek, synergy HT) at a wavelength of 580 nm. In this assay, the optical density is proportional to the number of viable (or metabolically active) cells in each well.

### 4.5. Colony Formation Assay

A colony formation assay was performed to assess the survival and clonogenic activity of cancer cells in response to MØ-derived factors and the influence of DAB-2-28 on this process [27]. Briefly, cells were seeded in 24-well plates (1.5 × 10^5^ cells/well) and incubated for 48 h in 500 μL of CM-Ctl or CM-MØ, diluted 1:3, in complete culture medium. After 48 h of incubation, cells were treated with vehicle (0.1% DMSO), DAB-2-28 (30 μM), or Cisplatin (15 μM) for 1 h and then harvested and counted. Treated cells were cultured in a 6-well plate (200 cells/well) and incubated for 21 days in complete culture medium (1.5 mL) to allow adhered cells to form colonies. After 3 weeks, colonies were fixed with formalin solution (10% *v*/*v*), stained with crystal violet solution (0.01% *v*/*v*), and then counted using a phase contrast optical microscope.

### 4.6. Induction of EMT in MCF-7 Cells

A protocol was developed to induce EMT in MCF-7 cells using macrophage-derived conditioned media and StemXvivo (#CCM017; EMT inducing media supplement, R&D Systems). StemXvivo is a culture medium supplement specifically designed to induce EMT in several cell lines [29], including MCF-7 cells. Thus, it was used as an alternative method to induce EMT to compare the effect of MØs-conditioned media. MCF-7 cells were cultured in 6-well plates (1.75 × 10^5^ cells/well) and incubated with 1.5 mL of CM-Ctl or CM-MØ, diluted 1:3, or StemXvivo and PBS, diluted 1:100, in culture medium containing 2.5% FBS, for 7 days, with replacement with a fresh dilution on day 4. The evolution of EMT in MCF-7 cells was checked during the experiment by monitoring cell morphology, and pictures were taken using phase contrast microscopy at 20× magnification. After 7 days, cells were harvested by trypsin treatment and lysed in TNE-based lysis buffer (10 mM Tris, 1 mM EDTA, 100 mM NaCl, pH 8.0) containing 0.1% (*v*/*v*) Triton X-100 and protease inhibitors. Then, the total concentrations of proteins in the cell lysates were measured by colorimetric assay (DC protein assay kit) before immunodetection of proteins of interest by Western blot.

### 4.7. Western Blot

Western blots were performed to confirm the induction of EMT in MCF-7 cells by CM-MØ and to conduct signaling studies in MCF-7 and MDA-MB-231 cells. Cell lysates were prepared differently depending on the experiment. Proteins in cell lysates were separated in a 10% polyacrylamide gel and then transferred to a PVDF membrane, as previously described. Primary antibodies against E-cadherin (#3195), Snail1 (#3879), MMP9 (#13667) and the total (t-) and phosphorylated (*p*-) forms of STAT3 (#4904/#9145), NFκB (#8242/#3033), AKT (#4691/#4060), SMAD2 (#5339/#3108) and CREB (#4820/#9198) were purchased from Cell Signaling Technology (Danvers, MA, USA), and β-actin (#A3854) from Sigma Chemical Company (Oakville, ON, Canada). The secondary antibody (#1706515; Anti-rabbit IgG, HRP-linked antibody) was purchased from Bio-Rad Laboratories (Mississauga, ON, Canada). The chemiluminescence solution (SuperSignal West Femto) used to detect the HRP signal was obtained from Thermo Fisher Scientific (Saint-Laurent, QC, Canada). The total forms of the targeted proteins and β-actin were used as reference points in densitometric analyses when calculating the relative expression levels of activated proteins.

#### 4.7.1. Cell Lysates to Verify EMT Induction

The preparation of cell lysates to verify E-Cad expression after incubation of the cells in CM-MØ is described above (Section 4.6). The same protocol was followed to verify E-Cad expression in cells incubated with the cytokines TNFα (25 ng/mL), TGFβ1 (5 ng/mL), or TNFα + TGFβ1, or with StemXvivo reagent (1:100). To verify Snail1 expression, cells were cultured in 24-well plates (2.0 × 10^5^ cells/well) and incubated with CM-Ctl, CM-MØ, PBS, TNFα (25 ng/mL), TGFβ1 (5 ng/mL), TNFα + TGFβ1, or StemXvivo for 6 h. To study MMP9 expression, cells were incubated with CM-Ctl and CM-MØ for 24 h. In some experiments, pretreatment with vehicle (0.1% DMSO) or 30 μM DAB-2-28 for 1 h was performed before incubation with MØ-derived conditioned media or cytokines.

#### 4.7.2. Cell Lysates for Signaling Studies

Cells were cultured in 24-well plates (2.0 × 10^5^ cells/well) for 24 h. Cells were then starved for 3 h in serum-free culture medium before pretreatment with vehicle (0.1% DMSO) or 30 μM DAB-2-28 for 1 h and stimulation with MØ-derived conditioned media, PBS, or StemXvivo for 15 min. Cells were directly recovered in 200 μL of lysis buffer heated to 95 °C and containing 1% (*v*/*v*) SDS and protease and phosphatase inhibitors. For the preparation of homogeneous cell lysates, β-mercaptoethanol at a final concentration of 5% (*v*/*v*) was added and the samples heated to 95 °C prior to WB analysis.

### 4.8. Immunofluorescence

The expression of the epithelial marker E-Cad and the conformation of actin filaments (phalloidin) were analyzed by immunofluorescence (IF) in MCF-7 cells. Cells (4 × 10^4^ cells/coverslip) were plated on sterile square coverslips placed at the bottom of wells of a 6-well plate. Coverslips were incubated with 1.5 mL of conditioned media of MØs (1:3), PBS, or StemXvivo (1:100), diluted in complete culture medium, for 7 days, with a change to fresh dilutions on day 4. After 7 days, cells were fixed on the coverslips with 10% formalin for 20 min at room temperature; this was followed by a PBS wash. Then, the coverslips were incubated with blocking buffer (1X PBS/5% goat serum/0.3% Triton X-100) for 1 h and washed again with PBS. The coverslips were incubated for 18 h with the primary antibody (anti-E-cadherin, 1:200) diluted in antibody dilution buffer (1X PBS/1% BSA/0.3% Triton) and then with the secondary antibody (Anti-rabbit IgG Alexa Fluor conjugate 488, CST #4412S, 1:1000), diluted in the same buffer, for 30 min. Visualization of actin filaments was performed by incubating cells fixed on the coverslips with the fluorochrome-coupled anti-phalloidin antibody (CST #12877S, 1:20), diluted in PBS, for 20 min. Finally, the coverslips were washed with PBS and slide-mounted with a solution containing DAPI (ProLong Gold Antifade; Cell Signaling Technology, Danvers, MA, USA) and images were captured with a Leica SP8 confocal microscope (Leica Microsystems Inc., Concord, ON, Canada).

### 4.9. Boyden Chamber Invasion Assay

The effect of DAB-2-28 on the invasive potential of MCF-7 and MDA-MB-231 cells stimulated by soluble MØ-derived factors was assessed using a Boyden chamber invasion assay (HTS Transwell System; from Corning, NY, USA), as previously described [38]. The Boyden chamber consists of an insert that fits inside the wells of a 24-well plate containing a polycarbonate filter with 0.4 μM pores. The filter was covered with a layer of BME (Cultrex Basement Membrane Extract, Trevigen, MD, USA) diluted 1:10 (*v*/*v*) in serum-free culture medium, which forms a natural extracellular matrix. The BME was left to solidify overnight in a 37 °C incubator, with 500 μL of serum-free medium in the lower chamber. The next day, MCF-7 or MDA-MB-231 cells (5 × 10^5^ cells in 100 mL of culture media), pretreated for 60 min with vehicle (DMSO 0.1%) or DAB-2-28 (30 μM), were deposited on the BME layer covering the filter, while non-activated and activated MØs (5 × 10^4^ cells) were placed at the bottom of the well below the insert. The cells were left to invade for 48 h, after which the top side of the transwell insert, containing the BME and the non-invasive cells, was wiped using a cotton swab. The invasive cells, which were found on the bottom side of the transwell’s polycarbonate membrane, were fixed using a 10% formalin solution. The membrane was then cut out of the transwell insert and mounted on a microslide, using the ProLong Gold Antifade mountant with DAPI (Cell Signaling Technology, Danvers, MA, USA). The slides were observed by fluorescent microscopy (63×) and five fields were chosen at random for each condition to count the number of invasive cells in each field. The results are presented in the form of a graph, representing the number of invasive cells per field, according to the treatment condition.

### 4.10. Migration Assay (Wound-Healing Assay)

The effect of DAB-2-28 on the migration potential of MCF-7 and MDA-MB-231 cells was studied using wound-healing assays. MCF-7 (1.5 × 10^5^ cells/well) and MDA-MB-231 (2 × 10^5^ cells/well) cells were cultured in a 24-well plate and allowed to adhere for 18 h. Then, for MCF-7 cells, the culture medium was replaced with 500 μL of diluted (1:3) CM-Ctl or CM-MØ for 48 h, and for MDA-MB-231 cells, with culture medium containing 25 ng/mL with IL6 for 24 h. When cells reached 70–80% confluency, they were treated with vehicle (DMSO 0.1%) or DAB-2-28 (30 μM) for 60 min. Afterwards, two horizontal wounds were formed using p200 pipette tips, and cell debris was removed by washing twice with HBSS. Pictures of the wounds were taken at t = 0 h and at t = 24 h. Wound areas were analyzed and quantified with ImageJ software (version 1.54p) to calculate the percentage of wound closure.

### 4.11. Gelatin Zymography

Gelatin zymography was performed to study the impact of DAB-2-28 on the activation levels of MMP9 proteases secreted by MCF-7 and MDA-MB-231 cancer cells in response to MØ-derived factors. First, the cells were cultured in 6-well plates (6.0 × 10^5^ cells/well) and allowed to adhere for 24 h. The next day, the culture medium was replaced with 1.5 mL of diluted (1:3) CM-Ctl or CM-MØ for 48 h. Then, the CM was removed and the cells were pretreated with vehicle (0.1% DMSO) or 30 μM DAB-2-28 for 1 h. The cells were then washed 3 times with HBSS and incubated with serum-free culture medium. At this step, it is important to wash the cells thoroughly with HBSS so that no trace of FBS remains, as it contains MMPs. After 24 h of incubation, the supernatant was harvested and centrifuged to precipitate cell debris. The proteins in the supernatant were assayed by colorimetry (DC protein assay kit) to analyze the same amount of protein per sample (3 μg of protein). The proteins from the samples were then fractionated on a 10% SDS-polyacrylamide gel containing 1% porcine gelatin. After separation, the acrylamide gel was recovered and washed twice (30 min) with washing buffer. The gel was then placed in an incubation buffer for 18 h in a 37 °C incubator, with shaking to allow the gelatinases to degrade the gelatin in the gel. The gel was finally stained with a Coomassie blue solution until it turned blue, then destained with a destaining solution until clear bands appeared. The destained bands corresponded to the locations where the gelatin had been degraded by the hydrolytic activity of MMP9, for which gelatin is the specific substrate. MMP9 was identified based on its molecular weight using the molecular weight marker as a reference point. The gel image was then captured using a white light transilluminator, allowing for better visualization of the degraded gelatin bands, which appeared as white bands against the blue background of the stained gel.

### 4.12. Statistical Analyses

Data obtained from the experiments are presented as means ± SEM. Statistical analyses were performed using Prism software, version 9.4 (GraphPad). Means were obtained from at least three independent experiments, and the difference between groups was analyzed using a one-way ANOVA followed by a Tukey post-test. Statistical differences were considered significant at a *p*-value < 0.05.

## Data Availability

The original contributions presented in this study are included in the article. Further inquiries can be directed to the corresponding author(s).

## Abbreviations

The following abbreviations are used in this manuscript:

AKT	Protein Kinase B
BC	Breast cancer
CM	Conditioned media
COX-2	Cyclooxygenase-2
CREB	cAMP response element-binding protein
Ctl	Control
DMSO	Dimethyl sulfoxide
E-Cad	E-cadherin
EMT	Epithelial–mesenchymal transition
IF	Immunofluorescence
IL6	Interleukin 6
iNOS	Inducible nitric oxide synthase
MØ	Macrophage
MMPs	Matrix metalloproteases
MTT	Tetrazolium 3,(4,5 dimethylthiazol-2-yl)-2,5-diphennyltetrazolium bromide
NFκB	Nuclear Factor kappa-light-chain-enhancer of activated B cells
PBS	Phosphate-buffered saline
PMA	Phorbol-12-myristate-13-acetate
SMAD	Mothers against decapentaplegic homolog 2
Snail1	Snail Family Transcriptional Repressor 1
STAT3	Signal Transducer and Activator of Transcription 3
TAMs	Tumor-associated macrophages
TGFβ1	Transforming growth factor beta 1
TNBC	Triple negative breast cancer
TNFα	Tumor necrosis factor alpha
Twist1	Twist-related protein 1
WB	Western blot
ZEB1	Zinc finger E-box binding homeobox 1
